# Cognitive Decline in Neuronal Aging and Alzheimer's Disease: Role of NMDA Receptors and Associated Proteins

**DOI:** 10.3389/fnins.2017.00626

**Published:** 2017-11-10

**Authors:** Jesús Avila, María Llorens-Martín, Noemí Pallas-Bazarra, Marta Bolós, Juan R. Perea, Alberto Rodríguez-Matellán, Félix Hernández

**Affiliations:** ^1^Centro de Biología Molecular Severo Ochoa, Consejo Superior de Investigaciones Científicas, Universidad Autonoma de Madrid (CSIC-UAM), Madrid, Spain; ^2^Centro de Investigación Biomédica en Red de Enfermedades Neurodegenerativas, Instituto de Salud Carlos III (ISCIII), Madrid, Spain

**Keywords:** tau proteins, neurotransmitter agents, dendritic spines, cognition, therapies

## Abstract

Molecular changes associated with neuronal aging lead to a decrease in cognitive capacity. Here we discuss these alterations at the level of brain regions, brain cells, and brain membrane and cytoskeletal proteins with an special focus in NMDA molecular changes through aging and its effect in cognitive decline and Alzheimer disease. Here, we propose that some neurodegenerative disorders, like Alzheimer's disease (AD), are characterized by an increase and acceleration of some of these changes.

## Introduction

Human development and maturation are characterized by various stages, the final one being aging. The different stages are characterized by different cellular and molecular changes. The changes that occur during the final phase may, in part, result from the accumulation of alterations that have taken place in previous phases.

Aging is influenced not only by the programmed developmental process from gestation through to the final stages of human life but also by the environment (see Figure 2 of reference Sharon et al., 2016). Some of the hallmarks of aging in peripheral tissues are also common to aged brain cells (Table 1). These include an increase in reactive oxygen species production, together with a decrease in the removal of these species (Espinet et al., 2015; Yuan et al., 2015; Zhang et al., 2016), mitochondrial alterations (Santos et al., 2013; He et al., 2016), and the deterioration of neuronal stem cells (Licht et al., 2016; Table 2). Recently, a growing amount of literature demonstrates that alterations in peripheral tissues affect brain aging, being an example the influence of the gut microbiome (Lustgarten, 2016; Schroeder and Backhed, 2016; Sharon et al., 2016).

**Table 1:** Some hallmarks of aging in peripheral tissues that are also present in brain tissue.

	**Hallmarks of aging in peripheral tissues**
1	Genomic instability
2	Epigenetic alterations
3	Decrease in growth factors
4	Mitochondrial dysfunction
5	Loss of proteostasis
6	Stem cell exhaustion
7	Cellular senescence

**Table 2:** Some hallmarks of brain aging.

	**Hallmarks for brain aging**
1	Neuron senescence
2	Microglia activation and senescence
3	Changes in spine plasticity
4	Cytoskeletal changes
5	Changes in the amount and localization of neurotransmitter receptors

All of these changes can favor the development of neurodegenerative diseases. Indeed, aging is the main risk for Alzheimer's disease (AD), and it has been proposed that therapies seeking to slow down aging may also delay the onset of this condition. An example are blood factors that are able to revitalize hippocampal function (Wyss-Coray, 2016; Castellano et al., 2017). In this review, we address the aging-dependent alterations of the morphology of neurons and glia (mainly microglia), of a cytoskeletal component (microtubules), and of a cytoskeletal microtubule-associated protein (tau), and how these changes contribute to aging-dependent cognitive decline. To this end, here we focus on neurons present in brain regions, such as the hippocampus and cortex, which are involved mainly in memory and learning.

## Aging in neurons

The main risk factor for several neurodegenerative disorders, including AD, is aging. However, these disorders can be triggered by inherited mutations, environmental factors, and somatic mutations in the cells present in the central nervous system (CNS) (see for example Gomez-Ramos et al., 2017; Hoch et al., 2017) or read the proposed unifying mechanism in neurodegeneration that involves DNA damage and DNA repair errors in aged neurons (Ross and Truant, 2017).

Neuron morphology is characterized by the presence of several short and wide cytoplasmic extensions (dendrites), which may have some protrusions (dendritic spines), and a long and thin cytoplasmatic extension (axon), which may be wrapped by some glia (oligodendrocytes) structures. At the cellular (cytoskeleton) level, neurons display an age-dependent reduction in microtubules (Cash et al., 2003). It has also been proposed that the actin cytoskeleton contributes to aging (Gourlay et al., 2004; Mattson and Magnus, 2006). At cellular-molecular level, neuronal aging can be visualized by mean of universal biomarkers of cell senescence (Evangelou et al., 2017), namely lipofuscin (a fluorescent aggregate of oxidized proteins, metals and lipids) (Jung et al., 2007) and β-galactosidase activity (Dimri et al., 1995; Munoz-Espin and Serrano, 2014).

A main feature related to brain aging is cognitive decline. Cognitive capacity has been related to neuron number and function. Humans have around 86 billion neurons (Herculano-Houzel, 2012), and this number decreases during aging as a result of various factors. Selective neuronal susceptibility due to calcium dysregulation, mitochondrial perturbations, lack of neurotrophic factors, and cytoskeletal disruption, among others, may account for this decrease (Mattson and Magnus, 2006). Thus, brain atrophy occurs during aging (O'Shea et al., 2016; Pini et al., 2016). A recent study indicates that two components related to neurodegenerative disorders, namely tau and amyloid beta (Aβ) peptide, are associated with memory encoding during normal aging (Marks et al., 2017).

Nevertheless, changes in neuronal function may occur prior to neurodegeneration as a result of a decrease in neuron-neuron connectivity through synapses. In this regard, analysis of such alterations is now unfeasible, given that it has been postulated that the number of synapses in humans could amount to around 11.5 × 10^14^ (Herculano-Houzel, 2012).

## Dendritic spines

There are several types of synapses, some are excitatory while others are inhibitory. The former can be identified on the basis of a spine-like shape, and since their discovery by Cajal they are referred to as dendritic spines (Ramon y Cajal, 1888 quoted in Yuste, 2015). The structure, dynamics and regulation of these spines are summarized in Hering and Sheng (2001).

**Figure 1:**
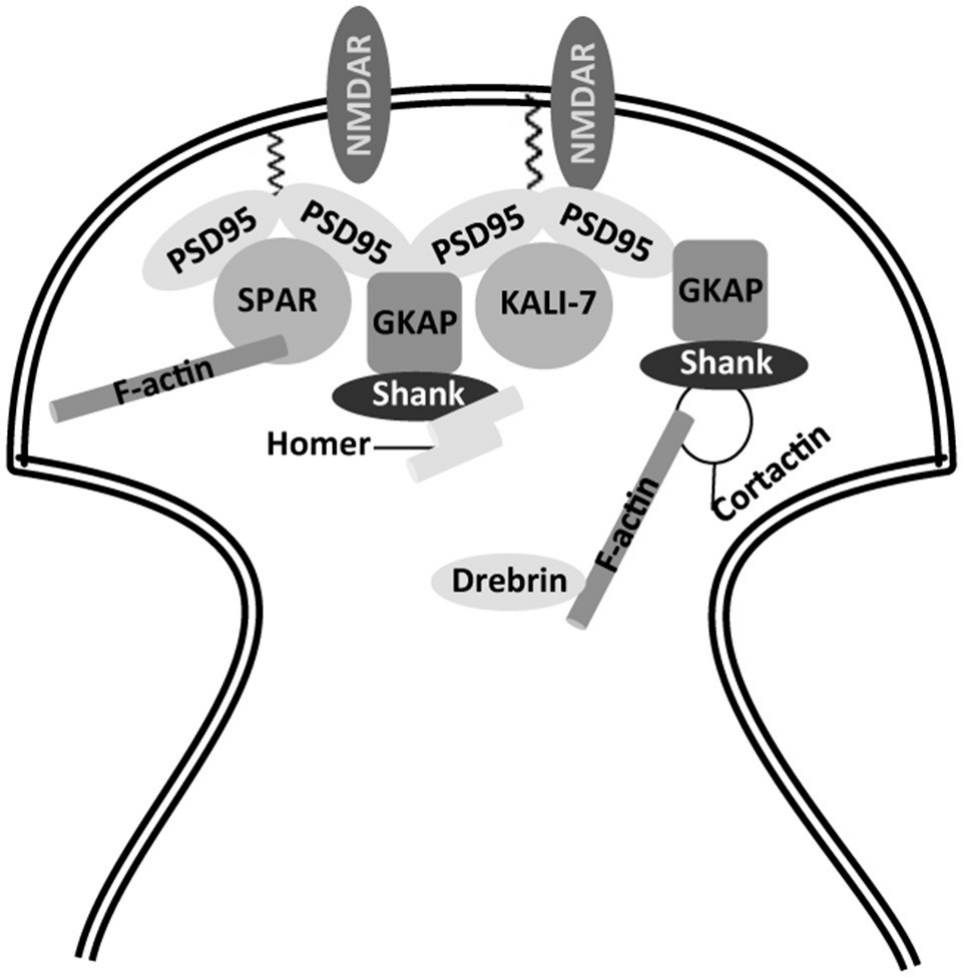
Partial view of actin cytoskeleton in dendritic spines. Scaffold proteins involved in anchoring of NMDA receptors to actin cytoskeleton. NMDAR, N-methyl-D-aspartate receptor; PSD-95, post-synaptic density protein 95; GKAP, guanylate kinase-associated protein; Shank, SH3 and ankyrin repeat-containing protein; SPAR, spine-associated RapGAP; KALI-7, kalirin-7.

The molecular scaffold of these spines is related to an actin cytoskeleton, composed of actin and actin binding proteins (Mattson and Magnus, 2006; Figure 1). Some of these proteins, like debrin (Hayashi and Shirao, 1999), bind to microtubule-binding proteins like EB3 (Dent, 2017). These in turn bind to other microtubule-binding proteins, like tau (Ramirez-Rios et al., 2016), a molecule that is also present in the spines (Ittner et al., 2010). Some of these proteins, together with small GTPases like Rac1 (Luo et al., 1996), RhoA (Mattson and Magnus, 2006), or SPAR (Naisbitt et al., 1999; Pak et al., 2001), regulate spine shape and function (Mattson and Magnus, 2006), through the formation of protein complexes with structural proteins like PSD95, Shank and Homer, among others (Naisbitt et al., 1999).

## Changes in dendritic spines with aging

**Figure 2:**
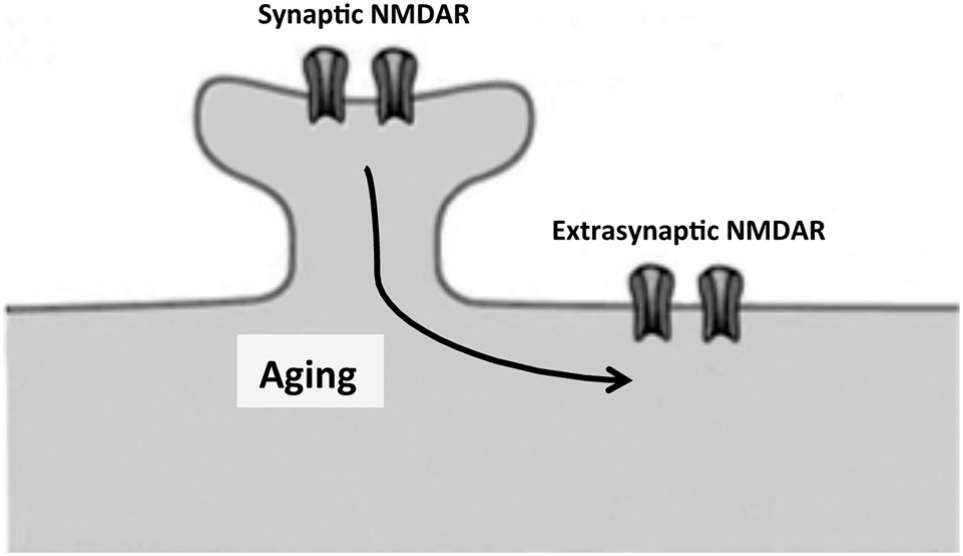
Proposed movement, by lateral diffusion, of NMDA receptors from dendritic spines to extrasynaptic sites. Unbalance between synaptic and extrasynaptic NMDAR may contribute to cognitive decline in neuronal aging and neurodegenerative diseases as Alzheimer disease.

Dendrites show progressive regression with increasing age in several brain regions (de Brabander et al., 1998; Kabaso et al., 2009). In a mouse model of aging, this regression occurs mainly in apical dendrites (Shimada et al., 2006). Glutamatergic receptors are among the key membrane proteins located on the surface of dendritic spines, and they participate in processes like learning and memory (Kumar, 2015). Glutamate acts on various membrane neuron receptors: NMDA, AMPA and ionotropic glutamate receptors (Dingledine et al., 1999; Conn et al., 2005). Here we will focus on NMDA receptors, which are found not only at the (synaptic) spine dendrites but also at extrasynaptic sites (Sun et al., 2016; Figure 2) although trafficking of AMPA receptors is also essential for synaptic plasticity and cognitive aging as well (Cantanelli et al., 2014).

## NMDA receptors

NMDA receptors are diverse in their subunit composition (GluN1, GluN2, and GluN3) (Paoletti et al., 2013). Combinations of GluN1 with a mixture of GluN2 or GluN3 subunits can build a functional NMDA tetramer (Paoletti et al., 2013). Four distinct GluN2 subunits (GluN2A, GluN2B, GluN2C, or GluN2D) can be present in this tetramer. Also, there are two distinct GluN3 subunits (GluN3A and GluN3B) (Paoletti et al., 2013).

GluN2 and GluN3 subunits differ in temporal expression. In the embryonic brain, GluN2B and GluN2D are present, while GluN2A and GluN2C expression starts after birth. After this point, GluN2D, and GluN2B expression decreases, the latter remaining mainly in the adult forebrain. GluN2C expression is found mainly in the cerebellum and olfactory bulb. In the case of GluN3 subunits, the expression of GluN3A occurs earlier than that of GluN3B, which is expressed mainly in motor neurons (for a comprehensive review on this subject, see reference Paoletti et al., 2013).

The function of GluN subunits may be related to their localization. GluN2A and GluN2B, present in hippocampus and cortex (Watanabe et al., 1993; Monyer et al., 1994; Laurie et al., 1997), have been associated with processes like learning and memory (Woodhall et al., 2001; Bidoret et al., 2009). Also, GluN2A, present in prefrontal cortex, may be required for working memory and its decrease is associated with age-related cognitive decline (McQuail et al., 2016). GluN2B appears to be crucial for channel function and post-synaptic macromolecular organization (Akashi et al., 2009). In the prefrontal cortex, this subunit may be involved in contextual fear memory (Zhao et al., 2005). In addition, GluN2B has been postulated to participate in depression (Tannenholz et al., 2016) and addictive behavior (Hopf, 2017).

NMDA receptors containing GluN2B are particularly mobile and segregate outside synapses to extrasynaptic sites (Triller and Choquet, 2005; Groc et al., 2006). This process may increase with aging (see below) (Figure 2).

## NMDA receptors during senescence

A possible relationship between impaired memory function and a decrease in NMDA receptors (Kumar, 2015) during senescence has been proposed. Thus, a decrease in NMDA receptor protein expression in regions like the hippocampus occurs during senescence (Magnusson, 1998). This decrease involve a reduction in GluN1 (Gazzaley et al., 1996; Liu et al., 2008). Also, an age-related decrease in the expression of GluN2A and GluN2B occurs in the hippocampus (Sonntag et al., 2000; Zhao et al., 2009). This decrease occurs together with a change in the localization of GluN2B from the synapse to extrasynaptic sites (Potier et al., 2010). A reduction in glutamate uptake has been associated with extrasynaptic NMDA receptors at the hippocampal CA1 synapse of aged rats (Potier et al., 2010). Recently, it has been reported that activation of extrasynaptic NMDA receptors induces tau overexpression (Sun et al., 2016). Since, the GluN2B subunit is present (Rammes et al., 2017) in extrasynaptic NMDA receptors, it has been considered a potential target for the treatment of neurodegenerative disorders related to aging, such as AD. In this context, it is especially interesting that in AD Aβ oligomers interact with the exposed regions of the subunit GluN1 (see for example Amar et al., 2017).

## NMDA receptor–tau interaction

**Figure 3:**
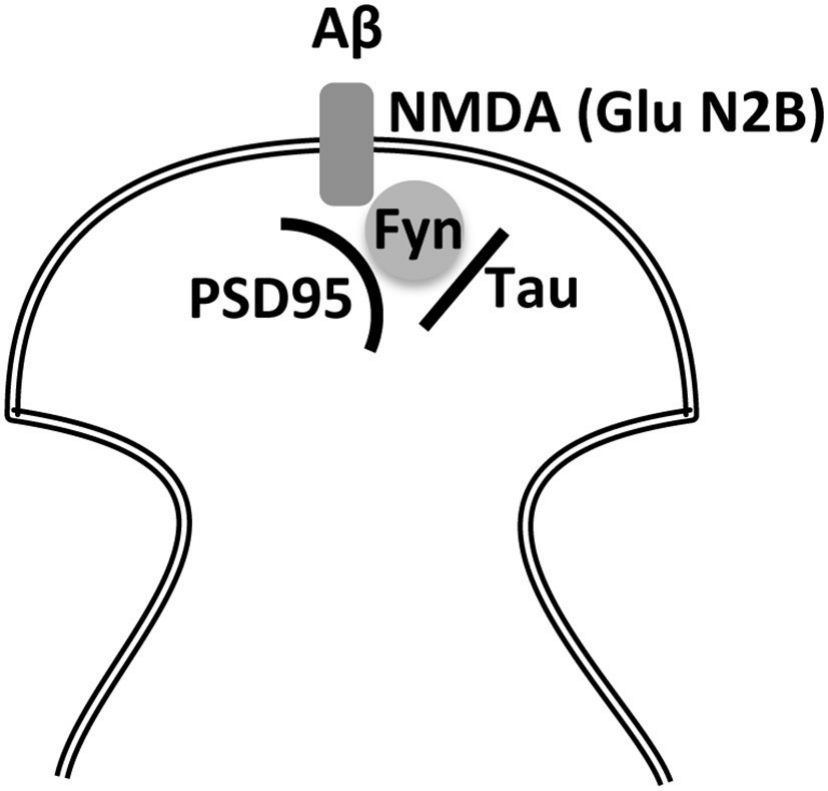
Indirect interaction between Aβ and tau through the NMDA receptor and fyn kinase. Two of the main molecular markers involved in Alzheimer disease, Aβ and tau, may require for their toxic effects of NMDAR-PSD-95 playing a role the kinase Fyn to alter post-synaptic density.

Synaptic GluN2B is phosphorylated by the tyrosine kinase fyn in a process regulated by tau (a protein present at dendritic spines Ittner et al., 2010). This phosphorylation is specific for this subunit and, upon phosphorylation, the NMDA receptor forms a complex with the post-synaptic density protein 95 (PSD95) (Ittner et al., 2010). Whether this complex favors the final morphology of dendritic spines remains unknown. However, the NMDA receptor-PSD95 interaction may be required for the toxic effect of Aβ peptide through its interaction with the NMDA receptor (Ittner et al., 2010), a toxic effect that could take place in AD (Figure 3). In addition, Aβ soluble oligomers (known as ADDLs) may interact with synaptic EphB2 receptors, proteins that are crucial for maintaining the integrity of NMDA receptors. Thus, loss of EphB2 mediated by ADDLs results in a decrease in surface localization of NMDA receptor subunits like GluN2B (Shi et al., 2016).

In hippocampal neurons, spines present at distal dendritic regions may have a larger window for long-term depression (LTD) than the proximal ones (Walker et al., 2017). Also, a decrease in the number of spines at distal dendritic regions in tau k.o. mice was found (Pallas-Bazarra et al., 2016). Taken together, these two results may explain in part the decrease in LTD found in tau k.o. animals (Regan et al., 2015). However, further research is needed to draw a clear conclusion since other factors, such as tau phosphorylation at Ser 396, are required for LTD (Regan et al., 2015).

## Tau and aging

The posttranslational modifications of tau, like phosphorylation, or its aggregation (Avila et al., 2013), can serve as a molecular marker of development, aging and neurodegenerative disorders (Hernandez et al., 2008). Also, a tau-like protein, present in C. *elegans*, could regulate neuronal integrity during aging (Chew et al., 2014).

Tau is a microtubule-associated protein and a microtubular reduction in this protein occurs in aging, as shown by analyzing pyramidal neurons of individuals of different ages (Cash et al., 2003). However, this reduction is not dependent on tau abnormalities that occur during aging, such as its aggregation (Cash et al., 2003), but on other unknown factors.

On the other hand, age-dependent changes in synaptic plasticity may enhance tau aggregation in mouse hippocampus (Kimura et al., 2017). Also, pathological aggregation of tau, in glia cells, could be a feature of aging in brain. An example is in aging-related tau astrogliopathy (ARTAG) (Liu et al., 2016).

## NMDA receptor–reelin interaction

Some proteins modify the age-dependent risk of cognitive impairment. One such protein, the apolipoliprotein isoform 4 (ApoE4), is a major risk factor for sporadic AD (Strittmatter et al., 1993). Furthermore, another protein, reelin, may exert a different role (Senkov et al., 2014). Both apoE and reelin share some cell receptors (Bal et al., 2013) and, one of them, ApoER2, appears to stimulate the coupling of the Dab1-Src/Fyn complex to the GluN2A and GluN2B subunits of the NMDA receptor, thereby facilitating the tyrosine phosphorylation of GluN2B (Doehner and Knuesel, 2010). A reduction of reelin expression during aging may contribute to cognitive impairment; however, appropriate reelin-mediated signaling may delay the shift to mainly pathological aging (Doehner and Knuesel, 2010). Of note, in AD, reelin expression is reduced in regions like the entorhinal cortex (Chin et al., 2007), which plays a role in cognitive capacity. Moreover, several relationships have been reported between reelin, the actin cytoskeleton, and dendrite spine growth (Chai et al., 2009; Caroni et al., 2014).

## NMDA receptor, microglia, dendritic spines and aging

In the aging brain, alterations occur not only in neurons but also in glia. Indeed, major shifts in glial regional identity are a transcriptional hallmark of aging in the human brain (Soreq et al., 2017). With respect to microglia, a link has been reported with the NMDA receptor. Microglia release D-serine, which may strengthen the synaptic response of NMDA receptor through the activation of its glycine site (Dhami et al., 2013). This process is altered in aged microglia (Hayashi et al., 2006). Furthermore, aging leads to impaired microglial function, which results in reduced brain resiliency, thereby increasing susceptibility to neurodegenerative diseases (Bickford et al., 2017).

However, a more relevant interaction takes place between microglia and dendritic spines. Microglia participate in the elimination of synapses—a process known as synaptic pruning (Paolicelli et al., 2011; Schafer et al., 2012), which takes place via complement activation (Hong et al., 2016; Lui et al., 2016). This and other microglia functions are altered with aging, thereby contributing to neurodegeneration as a function of age (Harry, 2013). Also, microglia show altered morphology and reduced arborization in the aged human brain (Davies et al., 2016). In addition, microglia transformation during aging results in changes in immune-modulatory functions of secreted factors showing a pro-inflammatory phenotype that favors neurodegeneration (Udeochu et al., 2016).

## Aging as a main risk factor for cognitive decline and dementia

The main risk factor for senile dementia like sporadic AD is aging. In fact, to study centenarians and their cognitive function would be a valuable manner to identify factors involved in healthy aging (Lavrencic et al., 2017). Despite neuronal death, AD is characterized by an increase in Aβ peptide, which could be toxic through its interaction (probably in oligomeric form) with a NMDA receptor subunit, GluN1 (Amar et al., 2017) present at the dendritic spines but also when it interacts with the glutamate receptor subunits present at extrasynaptic sites. The latter process of toxicity may involve tau protein in its modified form, which is also present in a higher proportion in the brains of AD patients. Figure 3 shows how these two molecules may exert a toxic effect on a dendritic spine. In this regard, Tyrosine kinase Fyn plays an important role (Ittner et al., 2010) by phosphorylating NMDA receptor subunit GluN2B. It could be postulated that, in the absence of this phosphorylation, the toxic effect of Aβ (which may occur in AD) will not take place. Little is known about the interaction of GluN2B with fyn-tau at extracellular synaptic sites and whether the presence of Aβ peptide has a toxic effect through its interaction at these sites by a mechanism involving the fyn-tau complex.

Table 2 shows some of the events that take place during aging and that are accelerated in neurological disorders like AD. These events include the following: neuron senescence in a hostile microglia environment; alterations in dendritic spines and in neurotransmission; and changes in the localization of neurotransmitter receptor from synapses to extrasynaptic sites. The latter alterations refer mainly to neurotransmitter receptors like NMDA receptors bearing a GluN2B subunit present at extrasynaptic sites, where they can interact with toxic ligands like Aβ peptide. The final result of the process at the functional level may be cognitive decline.

**Figure 4:**
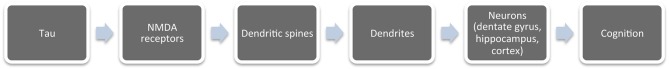
Different levels to study the changes that occur in the brain during aging. An alteration at any of these levels can cause cognitive impairment.

Therefore, changes in tau protein at the molecular level may contribute to the formation of protein complex (tau-fyn-PSD95-NMDAr). This complex may modify the shape or number of dendritic spines and/or the morphology of the neurons. Such alterations may lead to impaired neuronal function, thus promoting neurodegeneration (Figure 4).

## Therapies

Figure 4 indicates the different levels at which to analyze aging: the whole organism, brain regions, neurons, dendritic spines, NMDA receptors, and cytoskeleton, mainly tau proteins.

Adult hippocampal neurogenesis is linked to cognition and memory (Anacker and Hen, 2017). In the mouse, this process decreases with age (Sirerol-Piquer et al., 2011). It has recently been shown that treatment with Δ9-tetrahydrocannabinal (THC), a substance present in cannabis, enhances learning capacity and memory in aged mice (Bilkei-Gorzo et al., 2017). The administration of THC increases histone H3 and H4 acetylation at the klotho (an anti-aging protein) and BNDF promoters. Interestingly, a decrease in histone deacetylase HDAC3 improves memory capacity in older mice (Kwapis et al., 2017). In addition, the presence of some klotho fragments may enhance congnition in a mouse model (Leon et al., 2017).

On the other hand, transient overexpression of a negative regulator of dendritic spines, kruppel-like factor 9 (kef9), enhances the integration of newborn dentate granule cells into the neuronal network and may rejuvenate aged memory circuits (McAvoy et al., 2016). However, little is known about how to modulate the dynamics of dendritic spines and the role of microglia in synaptic pruning or in neuroinflamation (Ardestani et al., 2017).

Aging appears to be partly encoded in a blood-base signature, and it has been proposed that blood factors modulate aging and could find application for the rejuvenation of some organs, including brain (Wyss-Coray, 2016). The mechanisms of hippocampal aging and the potential for rejuvenation have been covered in an excellent review (Fan et al., 2017) and, recently, it has been reported that human umbilical cord plasma proteins revitalize hippocampal function in aged mice (Castellano et al., 2017). Indeed, the tissue inhibitor of metalloproteinase 2 (TIMP2), a factor in umbilical cord plasma, increases hippocampal-dependent cognition in these animals. At the neuronal level, it should be addressed whether abrogate senescent cells decrease aging in the surrounding cells (Baker et al., 2016). Also, it has been described that the presence of senescent cells contributes to tissue damage. A new technique through which to clear senescent cells without affecting non-senescent ones has been described (Baar et al., 2017). Brain regions, for example in the hippocampal zones CA1 and CA3, differ in their susceptibility to distinct components, such as zinc, which may affect subcellular structures like mitochondria (Medvedeva et al., 2017). In this regard, the chelation of zinc has been shown to enhance long-term potentiation in the CA1 neurons of aged rats (Shetty et al., 2017).

Also, an increase in growth factor expression could support neuron health. In this regard, methods to stimulate insulin production may prevent neuron aging (Hansen et al., 2015). In addition, the capacity of a modified peptide of the cilary neurotrophic factor to prevent synaptic deficits has also been tested with promising results (Baazaoui and Iqbal, 2017). Also recently, anti-aging strategies based on cellular reprograming have been tested in peripheral tissue (Ocampo et al., 2016). However, the effects of such strategies on neuronal tissue have not been addressed.

Little is known about how to modulate the dynamics of dendritic spines and the role of microglia in synaptic pruning or in neuroinflamation (Ardestani et al., 2017).

Also, effort should be channeled into the possible modulation of NMDA receptors subunits like GluN2B and their modification at tyrosine residues by fyn kinase. In this regard, memantine, an NMDA receptor antagonist, is currently used for the treatment of AD (Greig, 2015). Also, other NMDA receptor antagonists are under study (Raybuck et al., 2017).

Regarding the use of therapies targeting tau, mainly in modified forms, or Aβ in aging-related disorders like AD, there are abundant references. An example is a recent review that describes therapeutic strategies for restoring tau homeostasis to treat tauopathies like AD (Young et al., 2017).

In summary, the search for suitable treatments for aging-dependent cognitive decline continues at many levels.

## Author contributions

JA and FH: Conception and design, manuscript writing, editing and figure design. ML-M, NP-B, MB, JP, and AR-M: Manuscript writing, editing and synthesis of previous literature.

### Conflict of interest statement

The authors declare that the research was conducted in the absence of any commercial or financial relationships that could be construed as a potential conflict of interest.
